# Humanized Anti-RGMa Antibody Treatment Promotes Repair of Blood-Spinal Cord Barrier Under Autoimmune Encephalomyelitis in Mice

**DOI:** 10.3389/fimmu.2022.870126

**Published:** 2022-06-15

**Authors:** Takeshi Hirata, Takahide Itokazu, Atsushi Sasaki, Fuminori Sugihara, Toshihide Yamashita

**Affiliations:** ^1^ Department of Neuro-Medical Science, Graduate School of Medicine, Osaka University, Suita, Japan; ^2^ Sohyaku, Innovative Research Division, Mitsubishi Tanabe Pharma Corporation, Yokohama, Japan; ^3^ Department of Molecular Neuroscience, Graduate School of Medicine, Osaka University, Suita, Japan; ^4^ Central Instrumentation Laboratory, Research Institute for Microbial Diseases, Osaka University, Suita, Japan; ^5^ Department of Molecular Neuroscience, World Premier International Research Center Initiative (WPI)-Immunology Frontier Research Center, Osaka University, Suita, Japan

**Keywords:** multiple sclerosis, EAE, blood-spinal cord barrier, blood-brain barrier, vascular pathology, rgma, magnetic resonance imaging, neuropathology

## Abstract

The lack of established biomarkers which reflect dynamic neuropathological alterations in multiple sclerosis (MS) makes it difficult to determine the therapeutic response to the tested drugs and to identify the key biological process that mediates the beneficial effect of them. In the present study, we applied high-field MR imaging in locally-induced experimental autoimmune encephalomyelitis (EAE) mice to evaluate dynamic changes following treatment with a humanized anti-repulsive guidance molecule-a (RGMa) antibody, a potential drug for MS. Based on the longitudinal evaluation of various MRI parameters including white matter, axon, and myelin integrity as well as blood-spinal cord barrier (BSCB) disruption, anti-RGMa antibody treatment exhibited a strong and prompt therapeutic effect on the disrupted BSCB, which was paralleled by functional improvement. The antibody’s effect on BSCB repair was also suggested *via* GeneChip analysis. Moreover, immunohistochemical analysis revealed that EAE-induced vascular pathology which is characterized by aberrant thickening of endothelial cells and perivascular type I/IV collagen deposits were attenuated by anti-RGMa antibody treatment, further supporting the idea that the BSCB is one of the key therapeutic targets of anti-RGMa antibody. Importantly, the extent of BSCB disruption detected by MRI could predict late-phase demyelination, and the predictability of myelin integrity based on the extent of acute-phase BSCB disruption was compromised following anti-RGMa antibody treatment. These results strongly support the concept that longitudinal MRI with simultaneous DCE-MRI and DTI analysis can be used as an imaging biomarker and is useful for unbiased prioritization of the key biological process that mediates the therapeutic effect of tested drugs.

## Introduction

Multiple sclerosis (MS) is the most common demyelinating disease that can lead to severe as well as permanent sensorimotor deficits, and it currently remains incurable (1). Importantly, the clinical course of MS is highly variable, with frequency of relapse, severity of attacks, and time course of disease progression differing among patients (1, 2). Further, in some cases, patients suffer from progressive worsening of symptoms from the onset (referred to as primally progressive MS) (2). Therefore, the accurate clinical course description in each individual is critical for better prognostication and treatment decision-making (3). With regard to drug development, in pre-clinical studies, although it is desirable to determine a key mechanism of action of potential drugs *in vivo*, longitudinal and large- scale analysis of neuropathological alteration is still difficult. In clinical studies, the lack of appropriate prognostic, stratification, and surrogate markers complicates the sophisticated design of long-term clinical trials (4, 5).

In this respect, MRI is a promising tool for staging MS lesions and investigating the relationship between lesion outcomes and clinical manifestations (6, 7); for example, disease activity can be recognized by new gadolinium-enhancing lesions, or new/enlarging T2 lesions (6). In recent years, with the development of analytical technology, MRI is expected to be applied not only for evaluating disease activity but also for the assessment of remyelination by using diffusion tensor imaging (DTI) and magnetic transfer ratio. The possible utility of these parameters as outcome measures for testing drug efficacy has been previously reported (8, 9), however, it is not easy to validate the relationship between neurological symptoms and MRI parameters in clinical practice (5–7). As MRI can be used in rodents, it is possible to clarify the relationship among MRI parameters, neurological symptoms, and neuropathology by employing animal models of MS. However, evidence on the utility of MRI for the evaluation of therapeutic interventions in animal models is limited (10, 11).

Repulsive guidance molecule-a (RGMa) is a membrane-associated glycosylphosphatidylinositol-anchored glycoprotein that has been implicated in various central nervous system (CNS) disorders, including MS (12–14), spinal cord injury (15, 16), stroke (17), and neuromyelitis optica (18–20), both in experimental animal models (12–19) and in patients (12, 13, 16, 19, 20). In the case of MS, RGMa has been reported as upregulated in disease lesions (13), and in the experimental autoimmune encephalomyelitis (EAE) mouse model, anti-RGMa neutralizing antibody treatment was shown to attenuates motor symptoms by modulating T cell responses (12). RGMa-expressing dendritic cells can enhance T-cell activation, and when MOG-pulsed bone marrow–derived dendritic cells received RGMa silencing, their capacity to induce EAE following adoptive transfer to naive mice was diminished (12). By using a targeted spinal EAE model (21) in which a focal inflammatory lesion is induced, it has also been shown that anti-RGMa treatment promotes functional recovery (13, 14). Although the enhancement of axonal regeneration/rewiring and remyelination was considered as the underlying mechanism of motor recovery, it should be noted that functional recovery was observed promptly (within a few days) after anti-RGMa treatment (14), suggesting that other factors may exert beneficial effects in the early phase after treatment. However, previous evidence on the therapeutic effect of anti-RGMa treatment was mainly obtained *via* postmortem histological analysis, and information on the dynamic alteration in pathophysiology following treatment is currently lacking.

Thus, in this study, by applying high-field MR imaging in a locally-induced EAE (targeted EAE) mouse model, we aimed to evaluate the dynamic therapeutic profile of anti-RGMa antibody treatment. MRI analysis recapitulated the therapeutic effect of antibody treatment on the blood-spinal cord barrier (BSCB). The pronounced effect of anti-RGMa on the acceleration of BSCB repair was also confirmed *via* GeneChip analysis and immunohistochemistry (IHC). In addition, we also assessed the relationship between MRI parameters and neurological symptoms longitudinally, determining the characteristics at each time point. Furthermore, we found that the extent of BSCB disruption could be a highly predictive marker for myelin integrity in the later phase, thus proposing longitudinal MR imaging with simultaneous dynamic contrast-enhanced (DCE)-MRI and DTI analysis as an imaging biomarker for the assessment of therapeutic drug responses in MS.

## Methods

### Animals

Seven-week-old C57BL/6J female mice were purchased from Charles River Japan. Mice were born and raised under specific pathogen-free (SPF) conditions. Mice were housed in an air-conditioned room at 24 ± 2°C with a 12-hour light–dark cycle and had free access to water and food.

### Model Preparation and Treatment Protocol

Targeted EAE was induced in a manner similar to that previously described (14). Briefly, mice were immunized with MOG_35-55_ (200 μg, Sigma Genosys) and complete Freund’s adjuvant (2.5 mg, Chondrex). Three weeks later, after dorsal laminectomy at Th8, a 1.5 μL of cytokine mixture containing TNF-α (750 ng, R&D Systems) and IFN-γ (1 μg, Peprotech) was injected into the spinal cord at a depth of 0.5 mm. Pertussis toxin (List Biological Laboratories, Inc.) was administered intravenously 0 and 2 days post cytokine injection. Humanized anti-RGMa antibody (10 mg/kg) and an isotype control antibody (palivizumab, 10 mg/kg) were intravenously injected twice a week from 9 days post cytokine injection (DPI) onwards. Clinical scores were assessed based on the following criteria: 0, no abnormalities noted; 0.5, weak tail reflex; 1, loss of tail reflex; 1.5, loss of tail reflex with hind leg inhibition without gait abnormality; 2, partial hind limb paralysis with gait abnormality; 3, complete hind limb paralysis; 4, front and hind limb paralysis; and 5, moribund state. The assessor was blinded to the treatment groups.

### MR Measurement

**Figure 1:**
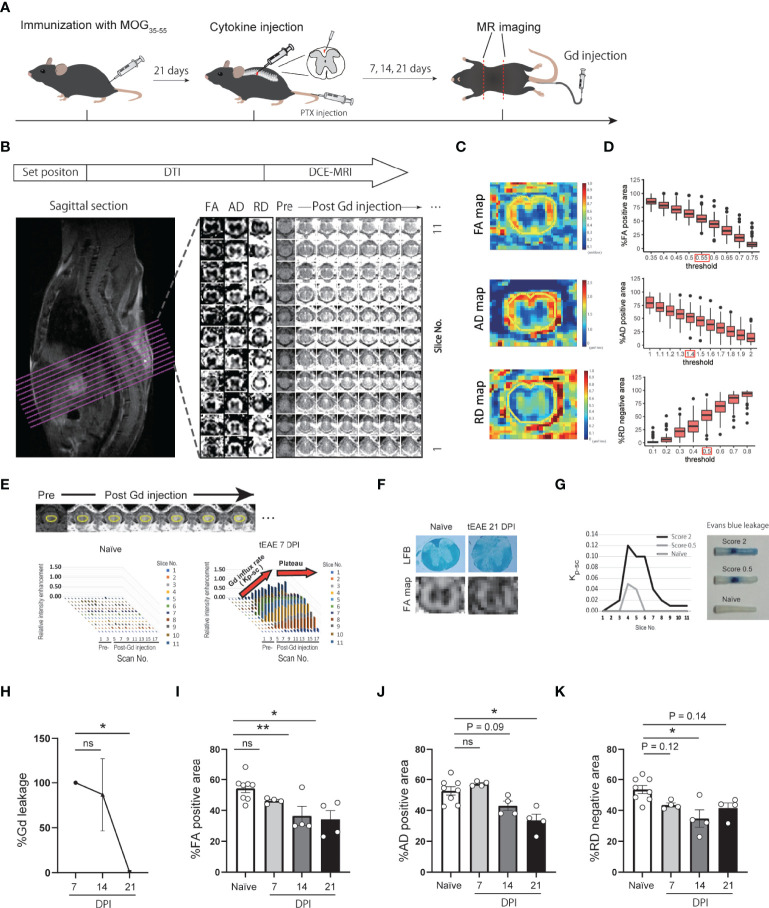
Development of DCE-MRI and DTI quantitative analytical method. **(A)** Graphical representation of MR imaging of targeted EAE mice. **(B)** MRI analytical flow. **(C)** Representative images of FA, AD, RD maps and ROI setting. **(D)** Histogram of %FA positive area, %AD positive area, and %RD positive area in each threshold. **(E)** Representative images of DCE-MRI and ROI setting (upper panel). Plots indicate relative intensity enhancement of pre- and post-Gd injection in naïve and targeted EAE mice at 7 DPI (lower panel). Gd influx into the spinal cord (Kp-sc) reach a plateau by 6 scans post-Gd injection. **(F)** FA map and LFB staining of spinal cord in naïve and targeted EAE mice at 21 DPI. **(G)** Kp-sc indicating the extent of Gd leakage into the spinal cord (left), and representative images of Evans Blue leakage into the spinal cord at 7 DPI (right) are presented. **(H)** Temporal change of the extent of Gd leakage. %Gd leakage at 14 and 21 DPI was calculated individually relative to the Gd leakage at 7 DPI. (n=4 for each timepoint, Dunnett’s multiple comparison test). **(I–K)** %FA positive area **(I)**, %AD positive area **(J)**, and %RD negative area **(K)** of naïve and targeted EAE mice at each timepoint (n=4, Dunnett’s multiple comparison test, compared to naïve mouse). Error bars represent the mean ± SEM. *p < 0.05, **p < 0.01. ns, not significant.

MR imaging were performed at 7, 14, 21 DPI (**Figure 1A**). A BioSpec 117/11 system (Bruker, 11.7 Tesla) equipped with Quadrature Volume Coils (Rapid Biomedical) was used for MR imaging. The fast spin echo protocol (RARE) was used for T1-weighted and diffusion tensor imaging (DTI). Sagittal T1-weighted imaging (TR/TE = 600/16 ms, 40 × 20 mm FoV, 256 × 128 acquisition matrix, 5 × 0.8 mm slices, 0.8 mm interslice distance, NEX = 8), axial T1-weighted imaging (TR/TE = 500/18 ms, 26 × 26 mm FoV, 200 × 200 acquisition matrix, 11 × 0.8 mm slices, 0.8 mm interslice distance, NEX =4), and DTI [single-shot echo-planar imaging with 6 diffusion directions (b = 4900 s/mm) and 1× b0 acquisitions, TR/TE = 2750/18 ms, 26 × 26 mm FoV, 128 × 128 acquisition matrix, 11 × 0.8 mm slices, 0.8 mm interslice distance] were conducted. The spinal segmental level was determined based on the vertebral landmarks, by using sagittal T1-weighted images (22).

DCE-MRI was performed using axial T1-weighted imaging. Following a baseline scan, Gd (Omniscan, Daiichi Sankyo, Japan) was injected as a bolus at 0.25 mmol/kg *via* catheter. A total of six axial images were obtained throughout 10 min following Gd injection. In both DTI and DCE-MRI, axial images were acquired to set the center of slice at 8th thoracic vertebra (**Figure 1B**).

### Image Processing and Calculation of MRI Parameters

The acquired images were analyzed using Fiji (http://fiji.sc/). Parametric images of DTI, fractional anisotropy (FA), axial diffusivity (AD), and radial diffusivity (RD) maps, which are considered as indicators of white matter, axon, and myelin integrity (23, 24), were used: especially, in EAE models, AD and RD maps were reported to correlates with axon integrity (confirmed by SMI staining), and myelin integrity (confirmed by MBP staining), respectively (23, 25, 26). Images were generated using built-in software (Paravision 5.1). The regions of interest (ROIs) in the slices were drawn around the spinal cords (**Figure 1C**). The FA, AD, and RD maps were binarized, and the area ratio to spinal cords was calculated as %FA positive area, %AD positive area, %RD negative area, respectively. The FA, AD, and RD binarized thresholds were determined to be ≥ 0.55 (unitless), ≥ 1.4 μm^2^/ms, and ≦0.5 μm^2^/ms, respectively. These thresholds were calculated so that the ratio of the binarized area to the whole spinal cord area was the same as that of white matter area to the whole spinal cord area, approximately 55% in the cytokine injection site, as shown in **Figure 1D**.

In DCE-MRI, ROIs were drawn in the spinal cords (**Figure 1E**). The extent of Gd leakage into the spinal cord was quantified as the sum of the Gd influx rate in each slice. The influx rate, Kp-sc (**Figure 1E**), was calculated as a method previously reported by Tatar et al. (27).

### Transcriptional Analysis of the Spinal Cord

Transcriptional analysis was performed at 14 DPI. Spinal cords were sampled with a width of 4.5 mm (centered on cytokine injection site) after transcardial perfusion with ice-cold PBS, then immediately frozen with liquid nitrogen and stored at −80°C until extraction. Total RNA extraction was performed using the Maxwell^®^ RSC simplyRNA Tissue Kit (Promega) according to the manufacturer’s protocol. Microarray analysis was performed using Mouse Genome 430 2.0 Array (Thermo Fisher Scientific) according to the manufacturer’s protocol. Single array analysis was performed using Microarray Suite version 5.0 (MAS5.0) and global scaling as the normalization method. Differential expression analysis and gene set enrichment analysis (GSEA) were performed using R-packages *limma* (28) and *clusterProfiler* (29) respectively. The gene set libraries for GSEA (**Supplementary File 1**) were prepared from Enrichr (30), CellMarker (31), and Munji et al. reported genes (32).

### Histological Analysis

Deeply anesthetized mice were transcardially perfused with ice-cold PBS, followed by 4% ice-cold paraformaldehyde (PFA) in PBS, then post-fixed overnight in 4% PFA in PBS. For Luxol Fast Blue staining, 10-μm transverse sections were incubated in 0.1% Luxol Fast Blue (Sigma) overnight at 56°C. Images were captured using an Olympus SZX16 bright-field microscope.

For immunohistochemistry, the spinal segmental level was determined based on the 8th thoracic vertebra, and the extracted spinal cord were divided into rostral, epicenter, and caudal segment of 3 mm each before post-fixation. Post-fixed samples were cryoprotected with 30% sucrose. Afterwards, spinal cord was serially sectioned into 20-μm transverse sections. Goat anti-P-selectin (1:1,000, AF737; R&D Systems), rabbit anti-collagen 1 (1:100, ab34710; Abcam), goat anti-collagen 4 (1:50, AB769; Sigma-Aldrich), rat anti-CD31 (1:25, 550274; BD Pharmingen), and goat anti-RGMa (1:100, AF2458; R&D Systems) were used as primary antibodies. For RGMa staining, antigen retrieval was performed by incubating the sections with 10 mM citrate buffer (pH 6.0) at 80°C for 10 min. Afterwards, sections were incubated with appropriate Alexa Fluor secondary antibodies (1:500, Invitrogen). Images were captured using an Olympus IX83 fluorescence microscope equipped with the CellSens software (Olympus).

To evaluate abnormal vascular lesions, CD31+, P-selectin+, perivascular collagen 1+, and collagen 4+ areas were measured. CD31+ vascular area was extracted using the canny edge detection and fill hole method, and the P-selectin high-intensity region within the CD31+ vascular area was calculated as the P-selectin+ area. The perivascular collagen area was calculated by subtracting the CD31+ area from the collagen+ area in vessels. All analyses were performed using MATLAB (MathWorks), and regions of interest were drawn using bright-field images. Three images were quantified for each spinal cord region, and the mean value of those was analyzed as individual animal data.

### Evans Blue Leakage

Evans Blue (Wako) dissolved in PBS (4% w/v) was intravenously injected at a total volume of 150 μL. After 1 h, the mice were transcardially perfused with ice-cold PBS, followed by 4% ice-cold paraformaldehyde (PFA) in PBS, under anesthesia. The leakage area at 7 DPI was evaluated to confirm its relationship with Gd leakage.

### Statistical Analysis

Dunnett’s multiple comparison test, Mann−Whitney test, one-way analysis of variance (ANOVA) followed by the Tukey–Kramer test, two-way ANOVA followed by the Bonferroni test, Pearson correlation analysis, Spearman rank correlation analysis, standardized partial multiple regression analysis, and simple linear regression analysis were performed using GraphPad Prism 9.0.2 (GraphPad Software). For transcriptional analysis, *p*-adjust value was calculated by the R-packages *limma* (28) and *clusterProfiler* (29). Error bars represent the standard error of the mean. *p* < 0.05 was considered to indicate statistical significance. For correlation analyses, the absolute value of correlation coefficients (*r*) was classified as follows: 0.0–0.2, very weak to negligible correlation; 0.2–0.4, weak correlation; 0.4–0.7, moderate correlation; 0.7–1.0, strong correlation. For regression analyses, the absolute value of R-squared (*r* ^2^) was classified as follows: 0.0-0.2, none or very weak effect size; 0.2-0.4, weak effect size; 0.4-0.7, moderate effect size; 0.7-1.0, strong effect size.

## Results

### Development of a Quantitative Method for DCE-MRI and DTI Analysis of Targeted EAE Mouse Spinal Cords

To evaluate the extent of BSCB disruption and myelin/axon integrity in the spinal cord of targeted EAE mice, we performed DCE-MRI and DTI by using high-field (11.7 Tesla) MRI (**Figures 1A–E**). To assess the validity of the DCE-MRI and DTI protocols, we compared fractional anisotropy (FA) maps with LFB staining for white matter integrity (**Figure 1F**), and the extent of Gd leakage with Evans blue leakage for BSCB disruption (**Figure 1G**). Both DTI and DCE-MRI reflected white matter integrity and the extent of BSCB disruption. We then performed longitudinal analysis of DCE-MRI and DTI at 7, 14, and 21 days post-induction (DPI) of targeted EAE. The extent of Gd leakage showed a peak in the acute phase (7 DPI) and then gradually decreased (**Figure 1H**). %FA positive area, %AD positive area, and %RD negative area exhibited a gradual decrease, suggesting the progression of white matter disintegration toward the chronic phase (**Figures 1I–K**; **Supplementary Figures S1A–D**).

### Humanized Anti-RGMa Antibody Ameliorates BSCB Disruption and White Matter Disintegration in Targeted EAE Mice

**Figure 2:**
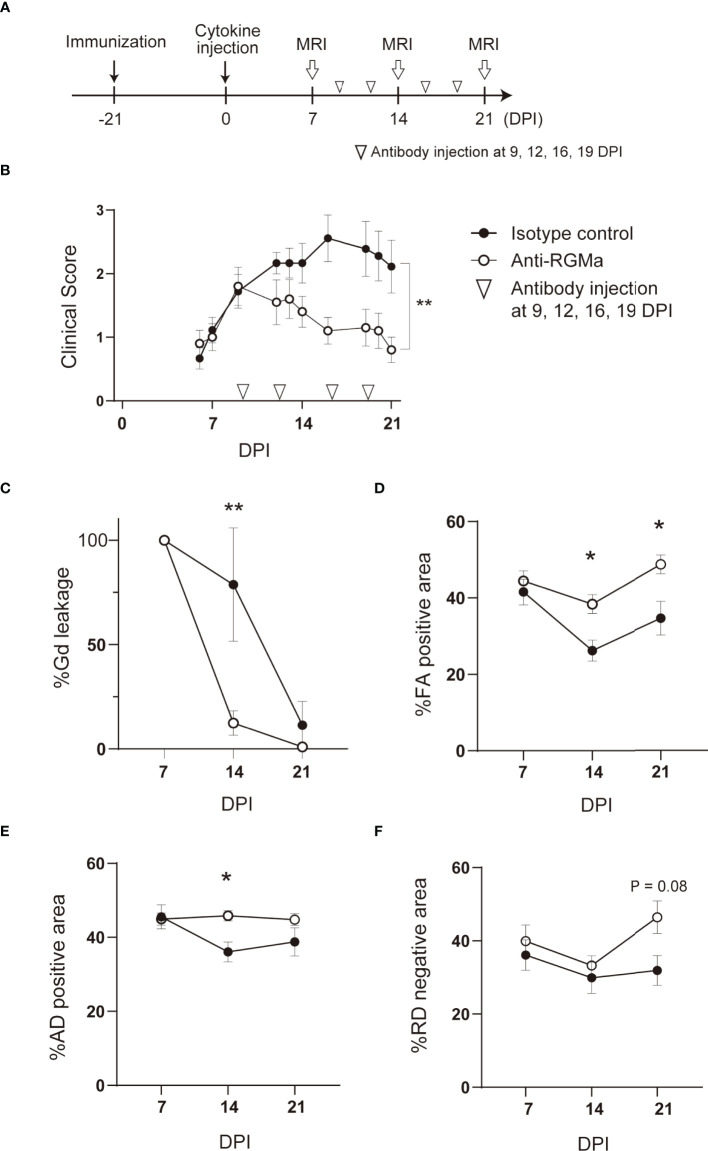
Humanized anti-RGMa antibody ameliorates the neurological symptoms and MRI parameters in targeted EAE mice **(A)** Experimental time course. **(B)** Anti-RGMa antibody treatment significantly improves clinical score (isotype control; n=9, anti-RGMa; n=10, Mann-Whitney test). **(C)** Anti-RGMa antibody attenuates %Gd leakage at 14 DPI (isotype control; n=8, anti-RGMa; n=9, two-way ANOVA followed by Bonferroni’s multiple comparison test). **(D–F)** Effect of anti-RGMa antibody on %FA positive area, %AD positive area, and %RD negative area (isotype control; n=9, anti-RGMa; n=10, two-way ANOVA followed by Bonferroni’s multiple comparison test). Error bars represent the mean ± SEM. *p < 0.05, **p < 0.01.

To evaluate the therapeutic effect of anti-RGMa, antibody administration was initiated after the onset of motor symptoms (9 DPI), while longitudinal DCE-MRI and DTI were performed at 7, 14, and 21 DPI (**Figure 2A**; **Supplementary Figures 1E–H**). In the preliminary experiments, we observed a tendency of worse outcome in individuals with a strong Gd leakage at 7 DPI. Therefore, to assess the effect of anti-RGMa antibody treatment, we divided mice into two groups (anti-RGMa antibody group and isotype control antibody group) based on the extent of Gd leakage at 7 DPI to avoid deviation between the groups. Anti-RGMa treatment had a prompt effect on clinical symptoms (**Figure 2B**). MRI parameters were also attenuated: at 14 DPI, the extent of Gd leakage was drastically reduced by the treatment, and the decreases in %FA positive area and %AD positive area were also attenuated (**Figures 2C–E**). Furthermore, both percentage areas were improved in the chronic phase (21 DPI) (**Figures 2D, F**). These results demonstrated that anti-RGMa antibody treatment had a strong and prompt therapeutic effect on BSCB disruption and white matter disintegration in targeted EAE mice.

### The Relationship Between Neurological Symptoms and MRI Parameters

**Figure 3:**
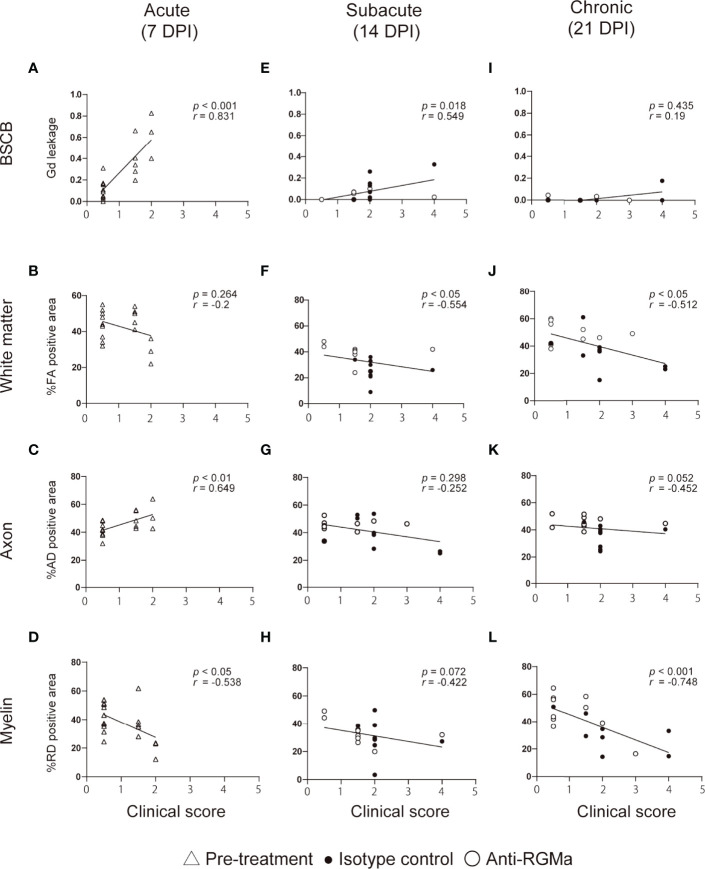
The correlation between clinical score and MRI parameters at each time point. Each plot shows individual values of clinical score and MRI parameters at 7 DPI (**A–D**, n=18-19), 14 DPI (**E–H**, isotype control; n=9, anti-RGMa; n=9-10), and 21 DPI (**I–L**, isotype control; n=9, anti-RGMa; n=9-10). The correlation coefficient and significance are expressed as *r* and *p*, respectively (Spearman’s correlation analysis).

To evaluate the correlation between each MRI parameter and neurological disability in targeted EAE mice at each time point, we performed Spearman’s correlation analysis (**Figure 3**). We also performed correlation analysis using pooled data from all timepoint (**Supplementary Figures 1I–L**). The extent of Gd leakage strongly correlated with the clinical score in the acute phase (7 DPI, *r* = 0.831, *p* < 0.001) and moderately correlated with that at the subacute phase (14 DPI, *r* = 0.549, *p* = 0.018). %RD negative area strongly correlated with the clinical score at the chronic phase (21 DPI, *r* = -0.748, *p* < 0.001). Furthermore, in order to evaluate the effect size of these MRI parameters on clinical scores, we calculated standardized partial regression coefficients (*β*) by using multiple linear regression analysis (**Table 1**). The extent of Gd leakage had a greater effect on the clinical score in the acute phase (7 DPI, *β* = 0.682, *p* < 0.001), while the %RD area had a greater effect on the clinical score in the chronic phase (21 DPI, *β* = -0.662, *p* = 0.039). Taken together, the extent of Gd leakage was highly correlated with neurologic disability in the acute phase, while the increase in RD was well correlated with that in the later phase.

**Table 1:** Multiple linear regression analysis for clinical score at each time point.

*Variable*	*Acute (7 DPI)*	*Subacute (14 DPI)*	*Chronic (21 DPI)*
Gd leakage	**0.682 (< 0.001)**	0.487 (0.147)	0.150 (0.482)
%FA	0.128 (0.585)	-0.106 (0.842)	0.136 (0.686)
%AD	0.264 (0.135)	0.0831 (0.823)	-0.235 (0.350)
*%RD*	*-0.141 (0.588)*	*-0.032 (0.941)*	***-0.662 (0.039)***

Data indicates standardized partial regression coefficient (p-value). DPI, day post cytokine injection; %FA, FA positive area ratio to spinal cord; %AD, AD positive area ratio to spinal cord; %RD, RD negative area to spinal cord. Bold values: p < 0.05.

### The Extent of BSCB Disruption Predicts Demyelination Severity

**Figure 4:**
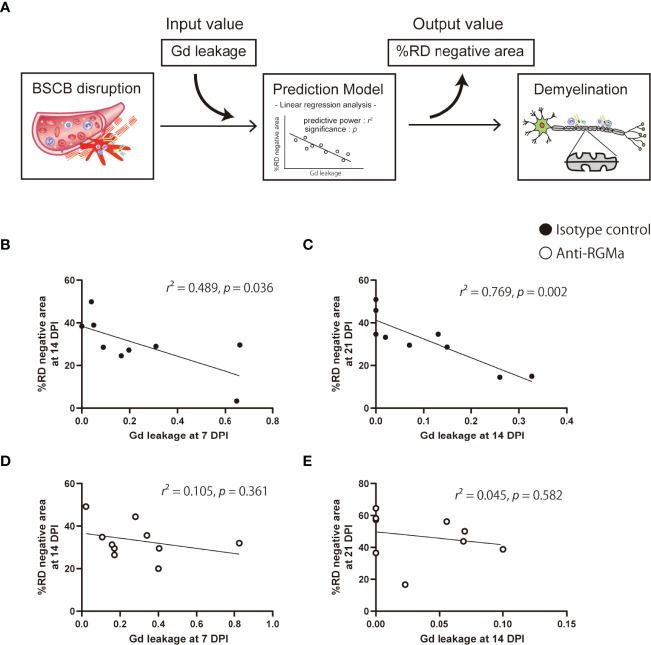
Prediction of myelin integrity based on BSCB disruption. **(A)** The prediction approach based on an input-output model with linear regression analysis. **(B–E)** Each plot shows the extent of Gd leakage and %RD negative area at 7 days in the isotype control group **(B, C)** and anti-RGMa group **(D, E)**. The effect size (*r* ^2^) of linear regression analysis and *p*-values are also shown.

To evaluate the strength of the relationship between the extent of BSCB disruption and neurological symptoms as well as white matter, axon, or myelin integrity, we performed correlation analysis between Gd leakage and the other parameters (%FA, %AD positive area, %RD negative area, and clinical score) at later time points based on data from the isotype control antibody group (**Table 2**). The extent of Gd leakage strongly correlated with %RD negative area 7 days later (Day 7 Gd leakage – Day 14%RD, *r* = 0.699, *p* = 0.036; Day 14 Gd leakage – Day 21%RD, *r* = 0.877, *p* = 0.002). Furthermore, in order to evaluate the predictive power of BSCB disruption with regard to myelin integrity after 7 days, we applied a prediction model based on BSCB disruption and demyelination. The extent of Gd leakage was used as the input value, %RD negative area at 7 days later was used as the output value, and the effect size (*r^2^*) of regression analysis was considered as predictive power (**Figure 4A**). In the isotype control group, the extent of Gd leakage predicted %RD negative area (moderately at 14 DPI, *r^2^* = 0.489, *p* = 0.036; strongly at 21 DPI, *r^2^* = 0.769, *p* = 0.002) (**Figures 4B, C**). However, anti-RGMa antibody treatment abolished its predictive power (14 DPI, *r^2^* = 0.105, *p* = 0.361; 21 DPI, *r^2^* = 0.045, *p* = 0.582) (**Figures 4D, E**). These results suggest that the extent of BSCB disruption can be used as a predictive biomarker for demyelination severity in the later phase.

**Table 2:** Correlation analysis between Gd leakage and MRI parameters in the control group.

		Gd leakage		Clinical score	
		7 DPI*^#^*	14 DPI*^#^*	7 DPI*^#^*	14 DPI*^#^*
14 DPI*^#^*	%FA	**-0.799 (0.010)**		-0.677 (0.056)	
	%AD	-0.162 (0.677)		-0.249 (0.524)	
	%RD	**-0.699 (0.036)**		-0.518 (0.167)	
	Clinical score	0.456 (0.278)		0.436 (0.500)	
21 DPI*^#^*	%FA	-0.271 (0.481)	-0.625 (0.072)	-0.438 (0.246)	-0.456 (0.278)
	%AD	-0.116 (0767)	-0.133 (0.734)	-0.398 (0.294)	-0.183 (0.778)
	%RD	-0.403 (0.282)	**-0.877 (0.002)**	-0.598 (0.095)	-0.639 (0.083)
	Clinical score	0.428 (0.275)	0.563 (0.133)	0.394 (0.345)	0.722 (0.056)

Data indicates Pearson’s and Spearman’s correlation (p-value). Spearman’s correlation analysis was used for clinical score correlation analysis. # DPI, day post cytokine injection; %FA, FA positive area ratio to spinal cord; %AD, AD positive area ratio to spinal cord; %RD, RD negative area to spinal cord. Bold values: p < 0.05.

### Transcriptional Analysis Shows the Effect of Anti-RGMa on BBB Dysfunction-Related Genes

**Figure 5:**
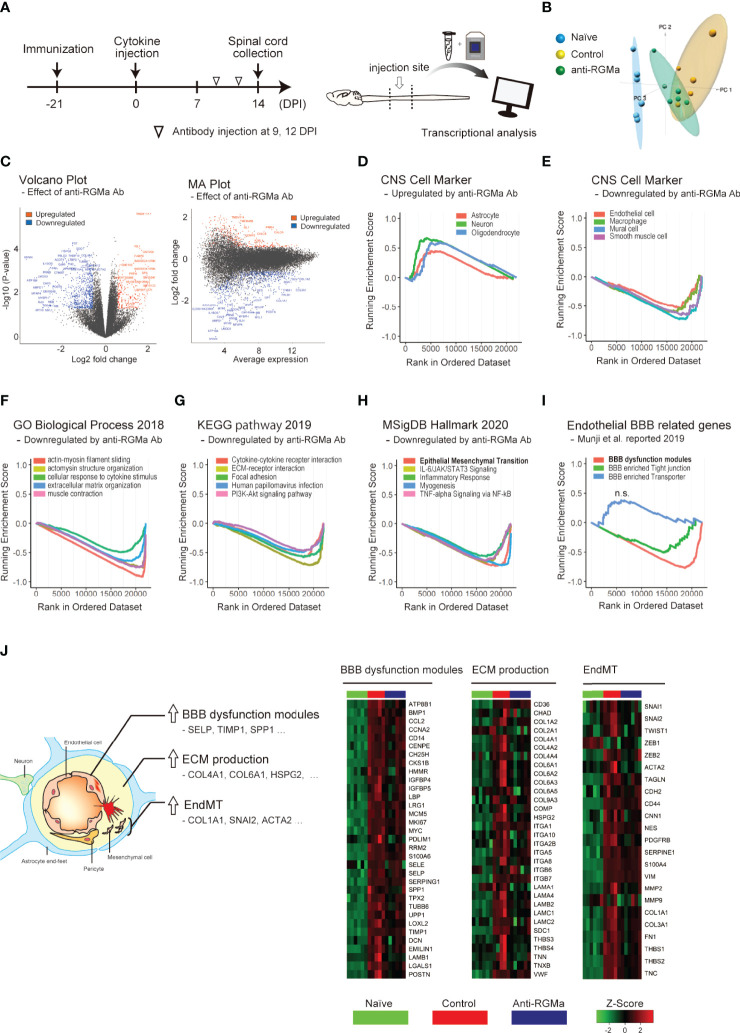
Transcriptional analysis of targeted EAE mice treated with control/anti-RGMa antibody. **(A)** Graphical representation of transcriptional analysis experiments. **(B)** Principal component analysis plot of each group. **(C)** Volcano plot (left) and MA plot (right) of the comparison between the isotype control group and the anti-RGMa antibody group. Differentially expressed genes (fold change>1.5, *p*-value < 0.05) were represented by color dots. **(D)** GSEA plot of CNS cell markers with positive enrichment in anti-RGMa antibody group compared to isotype control group (*p*-adjust < 0.05). **(E)** GSEA plot of CNS cell markers with negative enrichment in anti-RGMa antibody group compared to isotype control group (*p*-adjust < 0.05). **(F–H)** Top 5 GSEA plots with negative enrichment gene sets (*p*-adjust < 0.05) of GO biological process **(F)**, KEGG pathway **(G)**, and MsigDB hall mark **(H)**. **(I)** GSEA plot of BBB related genes sets (*p*-adjust < 0.05, n.s.; not significant). **(J)** Key features related to BBB disruption and corresponding genes (left) and heatmap of representative genes in BBB dysfunction modules, ECM production, and EndMT (right).

Given that anti-RGMa effectively ameliorated BSCB disruption 5 days after treatment initiation, we sought to explore the molecular profile involved in this effect. We performed microarray analysis of mouse spinal cords at 14 DPI (5 days after the first anti-RGMa antibody injection, **Figure 5A**). Using pre-processed microarray data (**Supplementary File 2**), principal component analysis was performed, and the result shows clear difference among naïve, control, and anti-RGMa antibody groups (**Figure 5B**). The differentially expressed genes (*p*-value < 0.05 and fold change > 1.5, isotype control group v.s. anti-RGMa antibody group) were presented in volcano plot and MA plot (**Figure 5C**), and the ranked gene list were used for GSEA. The GSEA results were summarized in **Supplementary File 3**. GSEA analysis of CNS cell markers showed significant enrichment of marker genes in neurons, oligodendrocytes, astrocytes, macrophages, mural cells, smooth muscle cells, and endothelial cells (**Figures 5D, E**; **Supplementary Figure 2**). In particular, endothelial cell and mural cell (pericyte) markers were negatively enriched in anti-RGMa treated group. Therefore, we further analyzed the gene sets with negative enrichment in GSEA (**Figures 5F-H**), and revealed that gene sets related to extracellular matrix production and epithelial mesenchymal transition (EMT) were strongly regulated by anti-RGMa antibody treatment. Further, we performed GSEA by utilizing endothelial BBB-related gene sets reported by Munji et al. (32), and revealed that anti-RGMa treatment significantly altered BBB dysfunction modules (**Figure 5I**; **Supplementary Figure 3**).

Alteration of gene expression in each gene set among the naïve, control antibody treated, and RGMa antibody treated groups is shown in **Figure 5J**. In addition to BBB dysfunction-related genes and ECM production-related genes, endothelial mesenchymal transition (EndMT)-related genes were also analyzed based on its similarity with EMT. Most genes in each group were upregulated by EAE induction, and this upregulation was ameliorated by anti-RGMa antibody treatment. Because EndMT is critically involved in endothelial dysfunction (33) including BBB pathophysiology (34, 35), these results support the therapeutic effect of anti-RGMa antibody on vascular pathology.

### Anti-RGMa Antibody Treatment Attenuates Vascular Pathology

**Figure 6:**
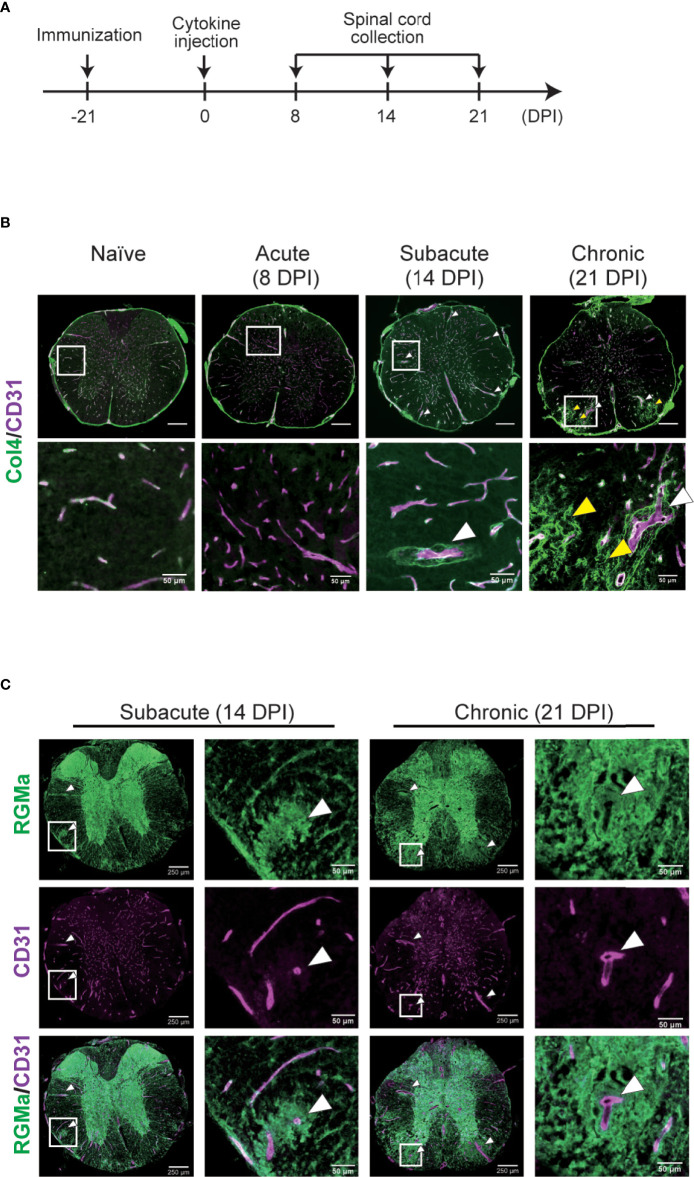
Immunohistochemical analysis of the spinal cord in targeted EAE mice. **(A)** Experimental time course of IHC analysis. **(B)** Representative image of the whole spinal cord stained with anti-CD31 and anti-type IV collagen antibodies at each disease phase in naïve mice and targeted EAE mice at 8, 14, 21 DPI. **(C)** Representative image of anti-RGMa and anti-CD31 antibodies staining in targeted EAE mouse spinal cords at 14 and 21 DPI. White and yellow arrows indicate abnormal vessels and collagen mesh formation, respectively. Scale bar; 50 μm (magnified), 250 μm (whole spinal cord).

**Figure 7:**
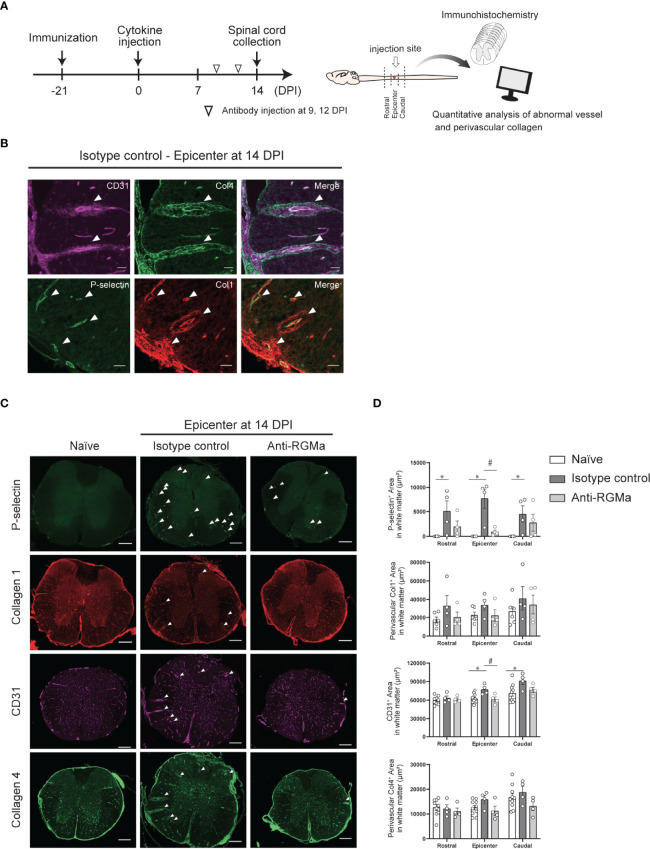
Immunohistochemical analysis of the spinal cord in targeted EAE mice treated by control/anti-RGMa antibody at 14 DPI. **(A)** Graphical representation of IHC experiments. **(B)** Representative images of vascular abnormalities at 14 DPI obtained from the isotype control group. **(C)** Representative images of the spinal cord stained for P-selectin, type I collagen, CD31, and type IV collagen in the naïve (left), isotype control (middle), and anti-RGMa antibody groups (right) at 14 DPI. Arrows indicate abnormal vessels. Scale bar; 50 μm (b, magnified image), 250 μm (c, whole spinal cord). **(D)** Quantification of P-selectin, CD31, perivascular type I and IV collagen-positive areas within white matter in each group (naïve; n=8, isotype control; n=4, anti-RGMa; n=4. Error bars represent the mean ± SEM. *p < 0.05 vs naïve, ^#^ *p <*0.05 vs isotype control, assessed *via* one-way ANOVA, followed by the Tukey-Kramer test).

To further confirm the effect of anti-RGMa on vascular pathology, we performed immunohistochemical analysis of vascular endothelial cell marker CD31 and basement membrane marker type IV collagen, both of which are BSCB components (**Figure 6A**). Endothelial cells exhibited abnormal thickening at 14 DPI (**Figure 6B**). Type IV collagen staining revealed a decrease in staining at 8 DPI, perivascular deposition at 14 DPI, and fibrous mesh formation at 21 DPI (**Figure 6B**). We also observed dense RGMa immunoreactivity around endothelial cells within the lesion site at 14 and 21 DPI (**Figure 6C**). Given the presence of remarkable vascular pathology at 14 DPI, we evaluated the effects of anti-RGMa antibody treatment (started at 9 DPI) by using P-selectin and type I collagen, which were included in the BBB dysfunction and EndMT-related genes and whose expression was altered by anti-RGMa based on transcriptome analysis (**Figure 7A**). As shown in **Figure 7B**, these proteins were confirmed as components of abnormal vessels in the spinal cord of targeted EAE mice. Subsequent analysis indicated that vascular abnormalities were prominent in the white matter, especially within the epicenter region (**Figure 7C**). Quantitative analysis revealed that perivascular collagen deposition in the white matter tended to be suppressed by anti-RGMa antibody treatment (**Figure 7D**). Moreover, endothelial cell abnormality and P-selectin upregulation were significantly attenuated by antibody treatment (**Figure 7D**).

## Discussion

In the present study, we revealed that BSCB repair-promoting effect is the key biological mechanism of action of anti-RGMa antibody treatment *in vivo* by applying a sequential MR imaging technique which enabled us to evaluate longitudinal changes in BSCB disruption, white matter integrity, axonal damage, and the extent of demyelination in targeted EAE mouse models. Importantly, the locally-induced spinal cord EAE model enabled us to avoid multiplicity, in time and space, of inflammatory lesions in the conventional EAE model, which makes it difficult to perform quantitative analysis of each imaging parameter in relation to neurological impairment. In our experimental approach, we successfully determined the relationship between MRI parameters and neurological symptoms as well as the key therapeutic effects of anti-RGMa antibody treatment.

To evaluate disease activity and pathological disintegration in targeted EAE mice over time, we sought to quantitatively investigate BSCB disruption as well as white matter, axonal, and myelin integrity *via* MRI (3, 8, 23–27, 36–39). For BSCB disruption, we analyzed the extent of Gd leakage into the spinal cord, while for white matter, axon, and myelin integrity, we calculated fractional anisotropy (FA), axial diffusivity (AD), and radial diffusivity (RD) in the white matter (8). Of note, although RD is considered a good indicator of demyelination/remyelination [21, 31-34], whether AD directly reflects axonal pathology is still under debate (8). However, as far as focusing on the acute stage of CNS injury, AD is expected to accurately characterize the extent of axonal degeneration (8).

We then investigated the relationship between MRI parameters and neurological symptoms longitudinally. First, we showed that the extent of Gd leakage gradually decreased from the acute to the later phase, while RD increased in the later phase, indicative of progressive demyelination. Such temporal transitions of EAE pathology are similar to those of clinical MS (1–3, 6). Moreover, in the acute phase, the extent of Gd leakage was highly correlated with neurological symptom severity, and, in the later phase, RD increase was well correlated with disability.

Importantly, we also found that the extent of Gd leakage could predict RD increase in the later phase (**Figure 4**; **Table 2**), that is, the extent of BSCB disruption detected by MRI could be used as a predictive marker for demyelination. Efforts to establish imaging biomarkers for therapeutic drug response evaluation are currently ongoing. However, reliable predictive markers have not yet been reported. Based on our results, by performing simultaneous DCE-MRI and DTI analysis longitudinally, it may be possible to determine the beneficial effect of pharmacological intervention through MRI. In particular, effective therapeutics can ameliorate the RD increase in the later phase (post-treatment) relative to the presumed RD value, which is predicted based on the extent of acute-phase (pre-treatment) BSCB disruption. This concept was further supported by our subsequent analysis of anti-RGMa antibody treatment.

Using our experimental method, we explored how spinal cord pathology is dynamically improved by humanized anti-RGMa antibody. Antibody treatment was initiated on day 9 after targeted EAE induction when motor deficits had already developed, and as expected, behavioral improvement was observed promptly after treatment initiation (**Figure 2A**). We revealed that anti-RGMa treatment drastically suppressed Gd influx into the spinal cord, as early as the first MRI session after treatment (5 days after treatment), which is strongly correlated with clinical score. FA and AD decrease, but not RD increase, were slightly attenuated, and at later time point (21DPI), RD increase exhibited a tendency to be suppressed. These results suggest that anti-RGMa exerts its beneficial effects by accelerating BSCB repair in the early phase, which may lead to the mitigation of axonal damage and the enhancement of remyelination. Importantly, the predictability of RD increase in the later phase based on acute-phase (pre-treatment) BSCB disruption level was compromised by anti-RGMa treatment. This strongly supports the notion that longitudinal MRI with simultaneous DCE-MRI and DTI analysis can be utilized as an imaging biomarker to determine therapeutic drug responses in MS.

With regard to the relationship between RGMa and the BBB, although it has been reported that RGMa silencing *via* an adenoviral vector improved BBB function in a rat MCAO/reperfusion model (40), whether RGMa inhibition has beneficial effects against an already-disrupted BBB/BSCB has not yet been investigated. Our results clearly indicated that delayed anti-RGMa antibody treatment still exerted a restorative effect on the disrupted BSCB, improving myelin integrity and motor function.

To further confirm the effect of anti-RGMa on the BSCB, we performed transcriptional analysis of the spinal cord 5 days after the first anti-RGMa administration. Based on GSEA analysis, endothelial cell marker and mural cell (pericyte) marker were negatively enriched in anti-RGMa treated group, and further analysis revealed that gene sets related to extracellular matrix production was strongly regulated by anti-RGMa antibody treatment. Moreover, EndMT related genes were also regulated by anti-RGMa antibody treatment. In the CNS, EndMT has been reported to be involved in pathological conditions by causing leaky vessel formation (34), and during MS pathophysiology, EndMT have been proposed to promote brain endothelial cell dysfunction.

Furthermore, we analyzed endothelial cell-related gene expression by utilizing a resource article that investigated BBB dysfunction modules (32), and found that most of the BBB dysfunction modules were attenuated *via* anti-RGMa treatment. Since the BBB dysfunction modules consist of common upregulated genes in four representative BBB disrupted disease models such as multiple sclerosis, seizure, stroke, and traumatic brain injury (32), it is noteworthy that the anti-RGMa treatment could accelerates BBB repair in various disease models.

Based on these results, we performed immunohistochemical analysis to confirm the effect of anti-RGMa on endothelial cells. In targeted EAE lesions, we observed a number of abnormal vessels which were characterized by aberrant thickening of endothelial cells, P-selectin upregulation, and perivascular type I and IV collagen deposits. Such vessels are frequently observed in both MS and EAE lesions (41, 42) and considered as the MS-related vascular pathology, and as expected, these pathological conditions were effectively attenuated by RGMa antibody treatment. These results further support the notion that the BBB/BSCB is one of the central therapeutic targets of anti-RGMa antibodies *in vivo*. Future studies are necessary to uncover the molecular mechanism of BBB/BSCB disruption and repair *via* RGMa-related signaling.

In the present study, we successfully evaluated the therapeutic effects of a humanized anti-RGMa antibody by using high-field MRI and proposed that the combination of DCE-MRI and DTI analysis can be used as an imaging biomarker for the evaluation of therapeutic interventions in MS. The current lack of representative biomarkers for therapeutic effect assessment is a serious obstacle for MS drug development, and quantitative imaging analysis is expected to solve these issues, especially in CNS disease (6, 7, 43, 44). As increasing evidence supports the validity of MRI parameters (including quantitative Gd leakage level and AD/RD) for evaluating MS in patients (37, 38, 45–55), our strategy should be further tested in clinical settings. In clinical MS, however, lesions can appear in a disseminated manner, which may complicate the application of our strategy, highlighting the need for a more detailed analytical protocol. Nonetheless, we believe that, in concept, establishing MRI-based predictive biomarkers, as proposed herein, is essential for sophisticated clinical trials. We also suggest that BSCB integrity is the key target of anti-RGMa treatment *in vivo*. Even in the BSCB-disrupted phase, anti-RGMa treatment drastically enhanced BBB repair in our experimental settings. For all previously reported animal models wherein anti-RGMa treatment had therapeutic effects, including spinal cord injury (15, 16), stroke (17), and neuromyelitis optica (18, 19), BBB/BSCB disruption is considered an important exacerbation factor. The BBB repair-enhancing effect of anti-RGMa may contribute to the amelioration of pathology in the above-mentioned models. As recent evidence has highlighted the importance of the BBB/BSCB in various CNS disorders, including neurodegenerative disorders (56), psychiatric disorders (57), and CNS complications of collagen-related diseases (58), it is of major interest to evaluate the effect of anti-RGMa for the treatment of these conditions in the future.

## Data Availability Statement

Genechip data was deposited in GEO database (GSE196545).

## Ethics Statement

All experimental procedures were reviewed and approved by the Institutional Animal Care Committee of Osaka University.

## Author Contributions

TY supervised the project. TI and TY conceived the study. TH performed most of the experiments. FS and AS provided advice on the methodology. TH, TI, AS, and TY analyzed the data. TH, TI, and TY wrote the manuscript with input from all authors. All authors contributed to the article and approved the submitted version.

## Funding

This work was supported by AMED-CREST (grant number 18gm1210005h0001) to TY.

## Conflict of Interest

TH and AS are employees of Mitsubishi Tanabe Pharma Corporation.

The remaining authors declare that the research was conducted in the absence of any commercial or financial relationships that could be construed as a potential conflict of interest.

## Publisher’s Note

All claims expressed in this article are solely those of the authors and do not necessarily represent those of their affiliated organizations, or those of the publisher, the editors and the reviewers. Any product that may be evaluated in this article, or claim that may be made by its manufacturer, is not guaranteed or endorsed by the publisher.

## Supplementary Material

Supplementary file 1 Gene set library for GSEA.

Supplementary file 2 pre-processed microarray data.

Supplementary file 3 GSEA results summary.

Supplementary Figure 1 MRI data related to -. **(A–H)** Temporal change of the MRI parameters of each mouse. (A-D) Non-treatment group (n=4). %Gd leakage **(A)**, %FA positive area **(B)**, %AD positive area **(C)**, and %RD negative area **(D)** are shown. Isotype control and anti-RGMa antibody treatment group (**E–H**, n=9-10). %Gd leakage **(E)**, %FA positive area **(F)**, %AD positive area **(G)**, and %RD negative area **(H)** are shown. **(I–L)** The correlation between clinical score and MRI parameters for all time point. Each plot shows individual values of clinical score and MRI parameters (pre-treatment; n=19, isotype control; n=18, anti-RGMa; n=20). %Gd leakage **(I)**, %FA positive area **(J)**, %AD positive area **(K)**, and %RD negative area **(L)** are shown. The correlation coefficient and significance are expressed as *r* and *p*, respectively (Spearman’s correlation analysis).

Supplementary Figure 2 Heatmaps of core enrichment cell marker genes. Heatmap of CNS cell markers which significantly enriched by anti-RGMa antibody treatment were shown. **(A)** Upregulated cell markers, **(B)** Downregulated cell markers of each group were expressed with Z score.

Supplementary Figure 3 Heatmaps of BBB enriched tight junction and transporter. Heatmap of BBB enriched Tight junction and transporter were shown. **(A)** BBB enriched tight junctions, **(B)** BBB enriched transporters of each group were expressed with Z score.
