# Supplementary figures and images for: Humanized Anti-RGMa Antibody Treatment Promotes Repair of Blood-Spinal Cord Barrier Under Autoimmune Encephalomyelitis in Mice
Source: Front Immunol. 2022 Jun 15;13:870126. doi: 10.3389/fimmu.2022.870126 (PMC9241446; doi:10.3389/fimmu.2022.870126)

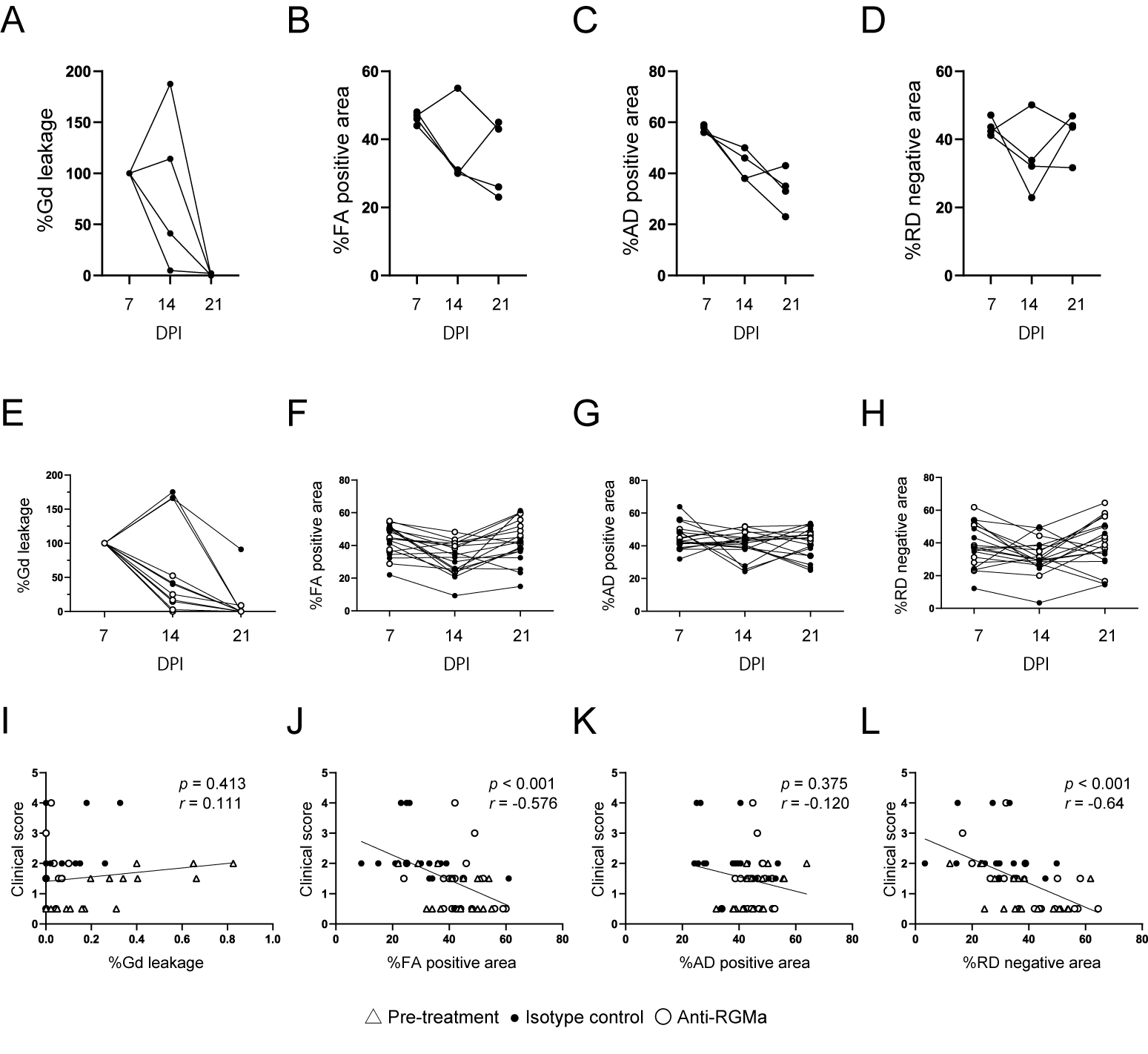

Supplement: Supplementary Figure 1 — MRI data related to -. (A–H) Temporal change of the MRI parameters of each mouse. (A-D) Non-treatment group (n=4). %Gd leakage (A), %FA positive area (B), %AD positive area (C), and %RD negative area (D) are shown. Isotype control and anti-RGMa antibody treatment group (E–H, n=9-10). %Gd leakage (E), %FA positive area (F), %AD positive area (G), and %RD negative area (H) are shown. (I–L) The correlation between clinical score and MRI parameters for all time point. Each plot shows individual values of clinical score and MRI parameters (pre-treatment; n=19, isotype control; n=18, anti-RGMa; n=20). %Gd leakage (I), %FA positive area (J), %AD positive area (K), and %RD negative area (L) are shown. The correlation coefficient and significance are expressed as r and p, respectively (Spearman’s correlation analysis). [file Image_1.tif]

A

CNS Cell Marker  
- Upregulated by anti-RGMA Ab

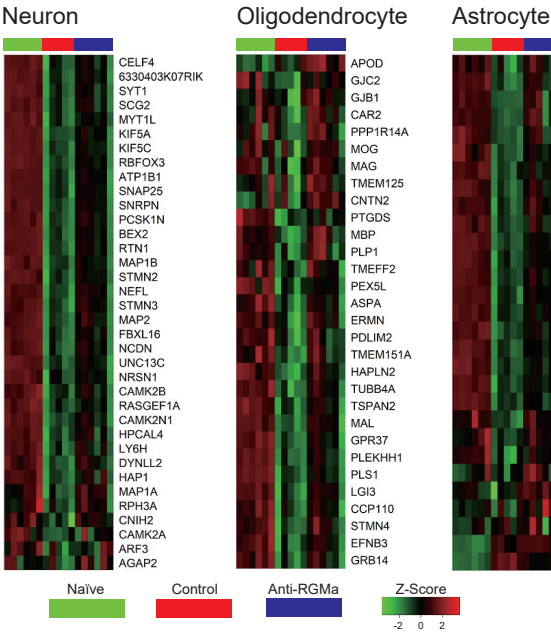

B

CNS Cell Marker  
- Downregulated by anti-RGMA Ab

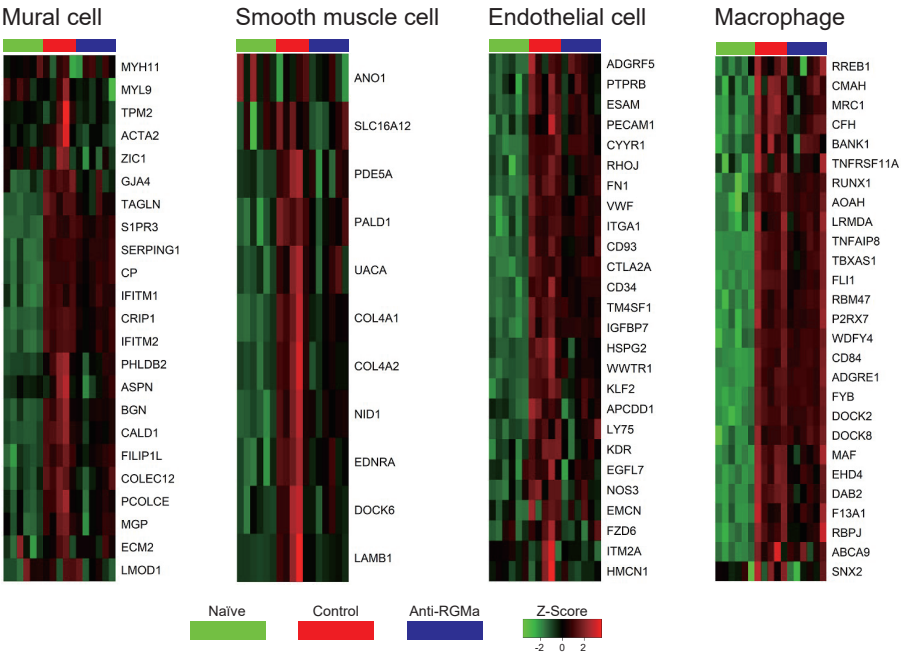

Supplement: Supplementary Figure 2 — Heatmaps of core enrichment cell marker genes. Heatmap of CNS cell markers which significantly enriched by anti-RGMa antibody treatment were shown. (A) Upregulated cell markers, (B) Downregulated cell markers of each group were expressed with Z score. [file Image_2.pdf]

A

## BBB enriched Tight junction

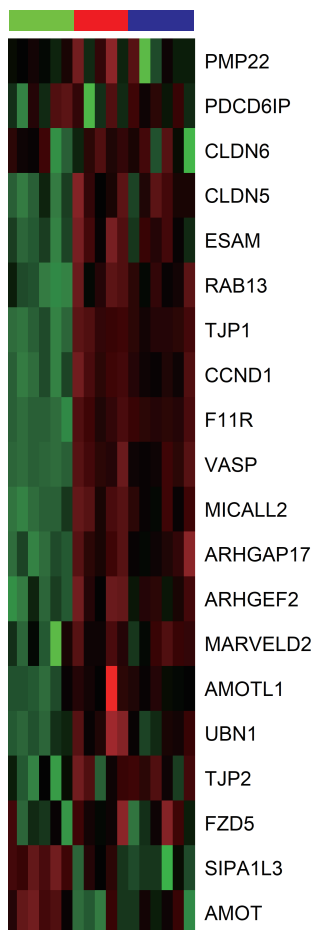

B

## BBB enriched Transporter

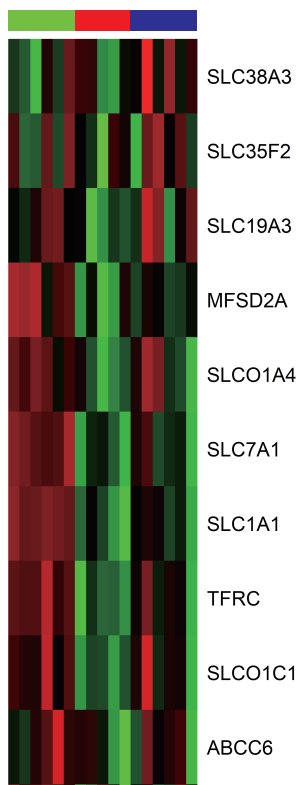

Z-Score

-2 0 2

Supplement: Supplementary Figure 3 — Heatmaps of BBB enriched tight junction and transporter. Heatmap of BBB enriched Tight junction and transporter were shown. (A) BBB enriched tight junctions, (B) BBB enriched transporters of each group were expressed with Z score. [file Image_3.pdf]
